# Treatment response assessment in mCRPC: is PSMA-PET/CT going to take the lead?

**DOI:** 10.1177/17588359241258367

**Published:** 2024-10-07

**Authors:** Martina Di Franco, Riccardo Mei, Camilo Garcia, Stefano Fanti

**Affiliations:** Nuclear Medicine, Alma Mater Studiorum University of Bologna, Via Massarenti 9, Bologna 40138, Italy; Nuclear Medicine Unit, University Hospital of Modena, Modena, Italy; Department of Nuclear Medicine, Gustave Roussy, Paris Saclay University, Villejuif, France; Nuclear Medicine, Alma Mater Studiorum University of Bologna, Bologna, Italy; Nuclear Medicine, IRCCS Azienda Ospedaliero Universitaria di Bologna, Bologna, Italy

**Keywords:** biomarkers, diagnosis, imaging, imaging biomarkers, PET guided treatment, prostate cancer, PSMA PET imaging, theranostics

## Abstract

The assessment of response to therapy in prostate cancer (PCa) patients is an ongoing, open issue. Prostate-specific antigen has limitations, especially in advanced metastatic PCa, which often displays intratumor variability in terms of response to therapy. Conventional imaging (i.e. computerized tomography and bone scan) is of limited use for its low sensitivity and specificity. Positron-emission tomography (PET) with prostate-specific membrane antigen (PSMA) demonstrated higher sensitivity and specificity, and novel PSMA-based criteria have been recently proposed for treatment response, with promising results in different scenarios, from chemotherapy to radioligand therapy. PSMA-based criteria have been found to outperform the current RECIST 1.1 and Prostate Cancer Working Group 3 frameworks in describing the behavior of PCa, precisely assessing tumor phenotypes through molecular-imaging-derived parameters. This review critically explores the current evidence about the role of PSMA PET/computed tomography in the assessment of treatment response.

## Introduction

Prostate cancer (PCa) ranks among the most common malignancies and death causes in males worldwide.^1^ The disease is characterized by a high percentage of relapse over the course of its natural history: more than 50% of individuals who undergo surgery or radiation therapy experience biochemical recurrence (BCR),^2^ eventually requiring additional treatments, mostly androgen deprivation therapy (ADT). Hormone-sensitive prostate cancer responds to ADT, but progression can ultimately occur despite low testosterone levels. Non-metastatic castration-resistant prostate cancer (nmCRPC) is diagnosed with a rising prostate-specific antigen (PSA) level without any sign of radiological progression, while metastatic castration-resistant prostate cancer (mCRPC) defines radiological evidence of metastatic progression.^3^

**Figure 1.**
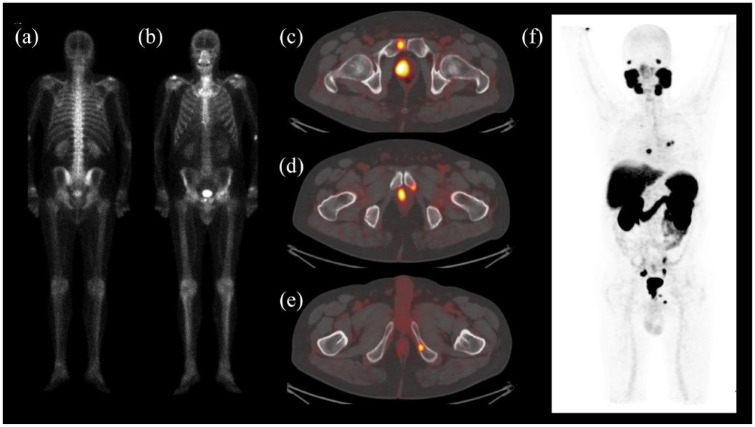
Bone scan (a, b) and [^68^Ga]PSMA PET/CT (c–f) performed for primary staging of a 64 year old patient with prostatic adenocarcinoma, Gleason score = 4 + 5, PSA = 10.1 ng/ml. The bone scan shows a small area of uptake at the right pubic bone (b). Fused transaxial images (c–e) and MIP (f) of the PSMA PET/CT scan confirm the right pubic bone lesion (c, f) and show further PSMA-positive bone lesions with intense PSMA uptake in the left pubic bone (d, f) and left ischio-pubic ramus (e, f). CT, computed tomography; MIP, maximum intensity projection; PET, positron-emission tomography; PSA, prostate-specific antigen; PSMA, prostate-specific membrane antigen.

Metastatic status in patients under ADT is generally identified by conventional imaging (CI), that is, computed tomography (CT) and bone scan,^4^ albeit with limitations including spatial resolution for the bone scan and poor characterization of small lymph nodes on CT^5^ (Figure 1). Moreover, bone marrow involvement can be missed using solely CI or hematological parameters.^6^

The epidemiology of PCa explains the efforts made over time to overcome these limits and improve diagnostic accuracy through novel imaging procedures.

The development of positron emission tomography (PET) radiopharmaceuticals targeting the prostate-specific membrane antigen (PSMA), typically overexpressed by PCa cells, has been a game-changer in the imaging field of PCa and in its therapeutic scenario over the last decade.

PET with CT imaging, performed using PSMA-binding radioligands, for example, among many [^68^Ga]Ga-PSMA-11 or [^18^F]F-PSMA-1007 (PSMA PET/CT), has been found to be more sensitive than CI,^5,7^ and it is recommended in the European guidelines for both staging high-risk patients and BCR.^8^ It is routinely employed in other clinical settings like staging unfavorable intermediate-risk patients, restaging nmCRPC, and in selecting patients eligible for radioligand therapy (RLT) with [^177^Lu]Lu-PSMA-617 (^177^Lu-PSMA).

mCRPC is known to be a heterogeneous group of diseases,^9^ often characterized by inter- and intratumor heterogeneity, due to which response assessment performed solely on the basis of PSA has been recently questioned.^10–12^ The higher detection accuracy of PSMA PET/CT compared to CI makes it suitable to be investigated for both early detection of metastatic spread and response assessment in patients with mCRPC.

Novel parameters and response criteria have been proposed over the past few years. Researchers hypothesize that PSMA PET/CT performed for response evaluation may provide an earlier and accurate definition of progressive disease (PD), which could be beneficial since novel biomarkers are needed to guide a tailored treatment for mCRPC patients.^13^

The purpose of this paper is to critically explore the current evidence about the role of PSMA PET/CT in the assessment of PCa treatment response, particularly in the setting of mCRPC.

## Methods

A search of the Medline database *via* Pubmed was undertaken to identify the most relevant findings on the role of PSMA/PET in PCa treatment response, stemming from articles published in the last 4–5 years.

## Recognizing mCRPC (are we doing it properly?)

Patients who are under ADT ultimately develop castration resistance.^3^

Castration-resistant PCa (CRPC) is defined by the presence of serum testosterone <50 ng/dl or 1.7 nmol/l plus, either, biochemical progression (three consecutive rises in PSA at least 1 week apart resulting in two 50% increases over the nadir and PSA >2 ng/ml) or radiological progression, if two or more new bone lesions on a bone scan or a soft tissue lesion occur, according to Prostate Cancer Working Group 3 (PCWG3) criteria.^4,8^

nmCRPC (biochemical progression only) and mCRPC (radiological progression) are often only temporally separated by the detection of new lesions by CT or bone scan, an event that affects one-third of CRPC patients within 2 years.^14^

The transition from nmCRPC to mCRPC is generally followed by a treatment switch, as androgen receptor pathway inhibitors (ARPI) alone are approved only for nmCRPC, on the basis of SPARTAN, PROSPER, and ARAMIS trials.^15–17^

Fendler *et al.* performed a PSMA-PET/CT scan to evaluate 200 patients with the same characteristics as the cohorts of the ARAMIS, PROSPER, and SPARTAN trials, that is, PSA doubling time ⩽10 months and/or Gleason Score (GS) ⩾ 8 (deemed to be at high risk for metastatic disease) without any signs of metastasis on CI. Of 200 patients, 44% exhibited PSMA-positive pelvic nodal disease, while 55% showed distant metastases; nearly all patients previously considered M0 had PSMA-detectable disease.^18^

Authors conclude that at the time of the recognition of metastatic status by bone scan or CT, a higher tumor burden has often been reached and that next-generation imaging could provide earlier recognition of low-volume mCRPC. This could influence further therapeutic decisions, for example, considering metastasis-directed therapy (MDT) or moving to systemic treatments. Moreover, for these patients, PSMA PET/CT might represent a baseline for any further response assessment.

However, the use of PSMA PET/CT in this setting can translate in what has been defined ‘up-staging’ the disease, that is, considering metastatic the same patients that according to CI would have been non-metastatic. In clinical practice, the potential up-staging by PSMA PET/CT would lead to a considerable drop-out of nmCRPC patients (according to CI) from treatment with ARPI as apalutamide or darolutamide, which are approved only for nmCRPC status.^15–17^

For this reason, changing therapeutic strategies on the basis of PSMA PET/CT is strongly argued and not recommended so far.^19^ Nevertheless, there is an increasing need to investigate the impact on overall survival (OS) of earlier and more accurate lesion detection, that is, identification of oligometastatic disease in CRPC.

## Oligometastatic mCRPC

Metastatic PCa management largely depends on previous treatments, and delaying next-line systemic therapies is fundamental, given the limited number of therapeutic options. To serve this role, MDT with surgery or stereotactic body radiotherapy (SBRT) has been proposed for PCa patients showing oligoprogression (the presence of up to three or five lesions, with no consensus on the maximum lesion number for defining oligometastatic disease), with promising results also in the CRPC subgroup.^20–23^ The studies are mostly retrospective and largely based on CI to determine oligometastatic status.^24^

The ORIOLE trial was a phase II randomized study that compared the efficacy of SBRT *versus* observation in hormone-sensitive PCa patients (*n* = 36 individuals in the study arm; *n* = 18 in the observation arm) in terms of progression at 6 months. Patients randomized to SBRT underwent baseline and post-treatment PSMA PET/CT scans, as a secondary goal was to examine the concordance between CI and PSMA PET through a blinded assessment. Seven out of 36 patients who received SBRT (19%) and 11 out of 18 patients in the observation arm (61%) experienced disease progression at 6 months (*p* = 0.005). The authors reported that of the 16 patients with PSMA-positive lesions at the baseline scan (untreated due to the blinded examination of PSMA imaging), 6 (38%) showed progression at 6 months, compared to 1 out of 19 patients with no untreated lesions (5%) (*p* = 0.03).^25^

Most studies examine the impact of MTD on hormone-sensitive PCa,^26,27^ and only few concern mCRPC.

A substantial limitation of the studies conducted so far is that researchers define oligometastatic disease by various image modalities, rarely including PSMA PET/CT.^28,29^ Moreover, the few that focus on PSMA PET/CT are based on the analysis of mixed populations of castration-sensitive and castration-resistant patients.^30–32^

In a retrospective study by Onal *et al*., 67 oligometastatic mCRPC patients studied with PSMA PET/CT and subsequently treated with SBRT reached a 2-year OS and progression-free survival (PFS) rates of 86.9% and 34.4%, respectively. Interestingly, the authors performed a PSMA PET/CT also to assess MDT response for the 32 patients without a PSA nadir after SBRT, documenting a complete metabolic response in 14 patients, partial response (PR) in 5, and PD in 13 of them. These results highlight the need of an early and precise restage in this setting, in order to eventually move forward to other therapies.^33^

**Figure 2.**
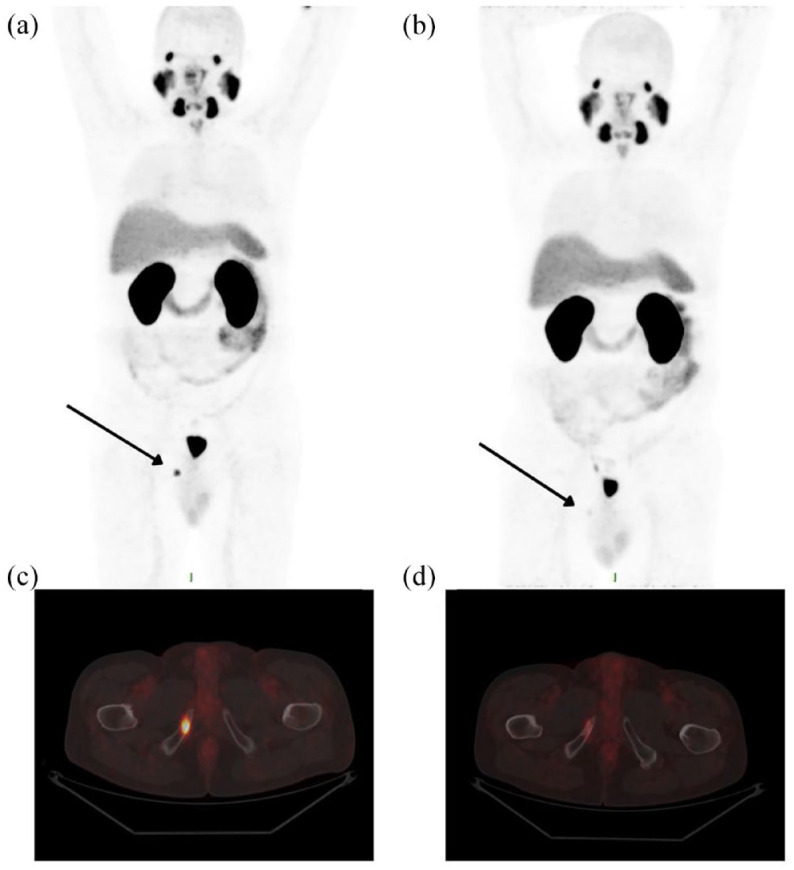
MIP (a, b) and fused PET/CT transaxial images (c, d) of a patient with mCRPC who underwent SBRT of the ischiopubic ramus. The bone lesion exhibits intense [^68^Ga]PSMA uptake on pre-SBRT PET scan (a, c) and a significant uptake decrease on post-SBRT PET scan (b, d). mCRPC, non-metastatic castration-resistant prostate cancer; MIP, maximum intensity projection; PET, positron emission tomography; PSMA, prostate-specific membrane antigen; SBRT, stereotactic body radiotherapy.

Figure 2 shows two PSMA PET/CT scans performed, respectively, before and after SBRT of a bone metastasis of the ischiopubic ramus in a patient with mCRPC. In this case, response to MDR, assessed by a PSA lowering, was complemented by a decrease in PSMA uptake.

Given the overall tendency of considering SBRT beneficial to delay next-line systemic therapies in oligometastatic CRPC, it could seem fare to demand high detection accuracy from imaging modalities. However, solid evidence of the impact of MDT on OS for this subgroup of patients is lacking.

Conclusive results from randomized controlled trials are needed to establish the role of PSMA PET/CT for patients with oligometastatic CRPC.

## PSMA PET/CT evaluation of response to systemic therapies in mCRPC and PSMA PET progression criteria

The range of systemic treatment options for mCRPC varies from next-generation androgen receptor-signaling inhibitors (abiraterone, enzalutamide), to chemotherapy (docetaxel and cabazitaxel), bone-targeted agents like [^223^Ra]Ra-dichloride (radium-223), and poly-ADP-ribose polymerase inhibitors, in cases of relevant genomic alterations in homologous recombination repair.^8^

The optimal sequencing of therapies depends on the previous treatments received: for patients who have received ADT alone (Luteinizing hormone-releasing hormone (LHRH) agonists, LHRH antagonists, or bilateral orchiectomy), ADT plus an ARPI, ADT plus docetaxel, or a triple combination of ADT + ARPI + docetaxel is possible.^8^

After a pretreatment with docetaxel, treatment options include cabazitaxel + prednisolone, abiraterone + prednisolone, or enzalutamide.^34–36 ^177^^Lu-PSMA therapy is considered for patients treated with at least one line of ARPI and one line of chemotherapy.^19^

The decision to switch from one line of therapy to another is driven by progression, which is currently established on the basis of PSA increase and the appearance of new lesions on a bone scan or CT, and RECIST 1.1, according to PCWG3 recommendations (Table 1).^4^

**Table 1.** Prostate Cancer Working Group 3 response assessment recommendations.

Variable	Criteria	
PSA	BR as ⩾50% decrease of baseline PD as ⩾25% increase and ⩾2 ng/ml above nadir SD as −50% < ∆%PSA < 25%	
Bone metastatic, using bone scan	For control/relieve/eliminate end points: - Resolved bone lesions - Improved or stable (no new lesions) - Worse (new lesions)	For delay/prevent end points (PD): - At least two new lesions on first post-treatment scan, with at least two additional lesions on the next scan (2 + 2 rule), which is confirmatory of progression - For scans after the first post treatment scan, at least two new lesions relative to the first post-treatment scan, confirmed on a subsequent scan
Nodes, using CT	Only report changes in lymph nodes that were ⩾1.5 cm in the short axis Record changes in pelvic (regional) nodes *versus* extrapelvic (distant/metastatic) nodes separately	
Visceral, using CT	Use Response Evaluation Criteria in Solid Tumors (RECIST) 1.1 with caveats: record changes in liver, lung, adrenal, and Central Nervous System separately. Only report changes ⩾1.0 cm in the longest dimension	

BR, biochemical response; CT, computed tomography; PD, progression; PSA, prostate-specific antigen; SD, stable disease.

The suggested earliest timing for measuring PSA levels is 12 weeks after the first cycle (a favorable effect on PSA may be delayed). In general, disease assessment according to PCWG3 should be performed at fixed intervals, that is, 8–9 weeks for the first 6 months and 12 weeks thereafter, but the optimal timing is not defined.

The limitations of this evaluation system range from the poor accuracy of CT and bone scans to the lack of consideration of heterogeneous responses and non-PSA-secreting disease.

The complexity of mCRPC reflects on its management, and decision-making processes require increasingly accurate acknowledgment of disease status, which is also needed for reproducibility in research methodology. The high positive predictive value and superior sensitivity of PSMA-PET/CT compared to CI are deemed to make it adequate for a more objective evaluation.^7,37^

Paller *et al.* encourage the use of PSMA as a marker of therapy response by analyzing the dynamics of PSMA expression on circulating tumor cells (CTC) during sequential therapies that included taxanes. By collecting peripheral blood samples of 96 patients over the course of at least two consecutive systemic treatments, they identified 15 men who had at least two CTC+ results. Using real-time PCR and gene expression assays, they evaluated PSMA expression on CTC as increased or decreased from the baseline. They observed a consistent and harmonious decrease in PSMA and PSA levels in responders, and a rise of both PSA and PSMA in non-responders. The authors concluded that the decline in PSMA, being proportional to cell number, can be explained by the elimination of PSMA-positive CTCs and not by downregulation of PSMA receptor expression.^38^

To examine a possible role for PSMA PET/CT-based evaluation of the response to chemotherapy, Seitz *et al.* investigated the concordance between biochemical response (BR) and radiographic response with [^68^Ga]Ga-PSMA PET/CT and CT separately in 16 mCRPC patients before and after three cycles of palliative docetaxel chemotherapy. The authors found concordance between BR and PSMA PET/CT response in 9 of 16 patients (56%), and between BR and CT response in only 4 of 12 patients (33%), suggesting that the [^68^Ga]Ga-PSMA PET approach might be superior to CT in response assessment.^39^

These results were confirmed also by Shagera *et al.* in their retrospective analysis of 29 mCRPC patients treated with taxane-based chemotherapy, with also a longer OS for PSMA responders than for PSMA non-responders. PSMA response was independently predictive of survival, whereas PSA could not significantly predict OS.^40^

PSMA overexpression in mCRPC, detected through PSMA PET/CT, has been demonstrated by other recent studies that documented the possible relationship between the variations in PSA serum levels and PSMA expression, evaluating its role in monitoring response to systemic therapies.^10,41–43^

Grubmüller *et al.* assessed treatment response to systemic therapies using [^68^Ga]Ga-PSMA-11 PET in 43 mCRPC patients undergoing 67 therapies (9 patients radium-223, 12 cabazitaxel, 22 docetaxel, 6 abiraterone, and 18 enzalutamide). The authors reported a significant association between all the PET parameters used and PSA response (∆total tumor volume *p* = 0.003, ∆ Standardized uptake value (SUV) mean *p* = 0.003, ∆SUVmax *p* = 0.011, ∆SUVpeak *p* < 0.001, ∆RECIST *p* = 0.012).^44^

Calderoni *et al.* conducted a retrospective analysis of 160 patients with mCRPC who were treated with various life-prolonging therapies, including ARPI, taxanes, radium-223, and ^177^Lu-PSMA. These patients underwent at least one PSMA PET/CT scan. The authors observed PSMA expression in 152 out of 160 (95%) patients. Additionally, they documented a 79% concordance between PSMA PET/CT and PSA response in cases where a second PET/CT scan was performed. PSMA response was determined based on the distinction between responders [cases of stable disease (SD), partial, or complete response] and non-responders (cases of progressing disease). The researchers reported that PSA change between the first and second PET/CT scans was +146% in non-responders and −57% in responders (*p* < 0.001).^43^

Plouznikoff *et al.*^42^ reported a total concordance between PSMA response and conventional response criteria in the subgroup of patients with mCRPC treated with enzalutamide and abiraterone (*p* = 0.006, Phi = 1 for enzalutamide; *p* = 0.001, Phi = 1 for abiraterone).

Despite the promising findings, so far there are no validated PSMA PET/CT-based response criteria.

The first PET scoring system was the European Organization for Research and Treatment of Cancer (EORTC) system, proposed in 1999^45^ and later overperformed by more practice-friendly PET Response Criteria in Solid Tumors (PERCIST) criteria in 2009.^46^

aPERCIST, adapting the FDG-based criteria PERCIST to PSMA PET/CT, are commonly used in PCa response assessment for the feasible application of thresholds and the use of target lesions, with progression defined as ⩾30% increase in summed SUVpeak and ⩾0.8 units relative to baseline measurements.

Alongside, PSMA-specific frameworks have been proposed.

PSMA PET progression (PPP) criteria,^47^ introduced by Fanti *et al*., first-time delineated specific parameters for PSMA-based progression assessment, combining variations in number of lesions, size, or PSMA uptake (by SUVmax), clinical and laboratory data, and changes in PSA levels before and after treatment (Table 2).

**Table 2.** PPP criteria.

Progression criterion
(a) Appearance of two or more new PSMA-positive distant lesions
(b) Appearance of one new PSMA-positive lesion plus consistent clinical or laboratory data and recommended confirmation by biopsy or correlative imaging within 3 months of PSMA PET
(c) No new lesions, but increase by ⩾30% in size or uptake plus consistent clinical or laboratory data and confirmation by biopsy or correlative imaging within 3 months of PSMA PET

PET, positron-emission tomography; PPP, PSMA PET progression; PSMA, prostate-specific membrane antigen.

PPP criteria are uncomplicated and reproducible, taking into account not only imaging data but also other commonly used parameters and providing a definition of progression that includes three different scenarios. Moreover, they offer prognostic information, distinguishing local from distant progression.

### Antiandrogen-induced PSMA upregulation and flare phenomena

Preclinical studies have documented an increase in PSMA expression produced by antiandrogen therapies through the action on *FOLH1* gene, containing the PSMA promoter and PSMA enhancer.^48^

Various studies have investigated PSMA modulation by ADT *in vivo*, with variable findings suggesting a possible PSMA upregulation early after ADT initiation in hormone-naïve patients, conceivably offset by a reduction in tumor size.^49,50^

Flare phenomena, meaning a persistence or enhancement of PSMA expression in PCa lesions, not related to progression, can lead to image misinterpretation. Therefore, the PCWG3 recommends the 2 + 2 rule for bone scan, that is, the appearance of two or more new lesions on the first post-treatment bone scan plus two additional lesions on the next scan.^4^ Moreover, it is recommended to avoid PSMA imaging within 3 months after the start of systemic therapy in hormone sensitive PCa.^51^

Regarding prolonged hormonal treatment, Afshar-Oromie *et al.* retrospectively analyzed the effect of long-term ADT on PSMA in 10 patients who underwent at least two PSMA PET/CT scans before and after ADT start (median ADT duration of 230 days before the second scan), and obtained PSA response. Of the 31 lesions detected at the baseline scan, 14 (45%) remained visible during ADT and 33.3% were visible even with complete PSA response in 6 patients. SUVmean increased in 4 lesions (12.9%), decreased in 22 (71%) and remained stable in 5 (16.1%).^52^

Impact of ADT on PSMA expression can differ between hormone sensitive and castration resistant patients with metastatic PCa, as described by Emmett *et al*., who performed serial PSMA PET/CT scans at baseline and after initiation of ADT in both groups. In 86.5% of hormone-sensitive patients (*n* = 8), SUVmax significantly decreased (median reduction of 30%) by day 9 after LHRH ± bicalutamide, along with PSA reduction (median −91%). Conversely, all castration-resistant patients (*n* = 7) showed increased SUVmax (median increase of 45%) by day 9 after starting abiraterone or enzalutamide, with a plateau at day 28, while PSA response was found in 5 of them. The authors hypothesize that PSMA receptors can be upregulated by ADT initiation in patients with mCRPC. Conversely, the PSMA decrease in the hormone-sensitive cohort can be explained by the elevated anti-proliferative action of first-line androgen blockade.^53^

Emerging therapeutic approaches include the combination between ^177^Lu-PSMA RLT and ARPI; therefore, a deeper knowledge of the mechanisms and timing of the interactions between antiandrogen therapies and PSMA could allow clinicians to decide the optimal schedule for PSMA imaging or even to benefit from PSMA upregulation, through the amplification of the target for RLT.

In conclusion, prior to using a PSMA PET/CT scan for response assessment purposes, the potential enhancing effects of hormonal therapy on PSMA expression have to be considered.

## PSMA PET/CT response assessment of ^177^Lu-PSMA therapy and RECIP criteria

PSMA-directed RLT consists of systemic administration of a low molecular weight PSMA-ligand labeled with beta-minus (β−) emitting isotopes (Lutetium-177 or yttrium-90) or alpha (α) emitting isotope actinium-225, for the purpose of delivering cytotoxic radiation to PCa cells overexpressing PSMA.^54^

[^177^Lu]Lu-PSMA-617 has been recently approved by Food and Drug Administration (FDA). The results from the randomized phase II TheraP trial suggested its superiority over cabazitaxel in terms of PSA response, adverse events, and PFS^55^; in the phase III VISION trial, treatment with [^177^Lu]Lu-PSMA-617 plus standard of care resulted in longer imaging-based PFS and OS compared to standard of care alone in mCRPC patients.^56^

The recent approval of [^177^Lu]Lu-PSMA-617 under the commercial name Pluvicto, by the FDA on 23 March and the European Medicines Agency (EMA) on 12 September 2022,^57,58^ comes with some open issues regarding response evaluation.

Currently, response assessment of PSMA RLT is based on serum PSA variations and CI, according to RECIST 1.1 and PCWG3,^4^ as for the other third-line treatments.

This system may no longer be appropriate due to the nature of mCRPC disease, which exhibits peculiar intra-tumor heterogeneity, often translating into mixed responses in terms of tumor size variations and the appearance of new sites.^9^ Consequently, CI and PSA dynamics cannot always reflect the course of the disease, also considering the possible occurrence of PSA-non-secreting lesions.^59^

Therefore, additional modalities for treatment response are under investigation.

Some studies underlined the possible application of ^177^Lu-PSMA Single Photon Emission Computed Tomography (SPECT/CT) and dosimetry in predicting the clinical outcome of patients undergoing RLT.^60,61^

In a study by Violet *et al*., dosimetric data, that is, whole body tumor dose, and SUVmean of the whole body tumor on the screening PSMA PET/CT were associated with PSA response at 12 weeks in a cohort of 30 patients with PCa who received ^177^Lu-PSMA. The authors found a significant correlation between PSMA PET/CT whole body tumor SUVmean and whole body dose.^62^

Pathmanadavel *et al.* performed quantitative analysis of ^177^Lu-PSMA SPECT/CT after the first and third cycles of ^177^Lu-PSMA RLT. They found that a 30% increased SPECT total tumor volume between baseline and week 12 was associated to a significantly shorter PSA PFS, thus predicting disease progression.^63^

SPECT/CT is feasible, cost-effective, and provides information that can be associated with clinical outcome in patients undergoing ^177^Lu-PSMA RLT. Its possible role in assessing RLT responses has to be further investigated.

On the other hand, PSMA PET/CT, which is mandatory for the selection of candidates for RLT, allows for the extention of lesion-based information that has been considered to fulfill the need. PSMA-based volumetric tumor parameters have indeed been recently studied^64–67^ and embedded in novel frameworks for response evaluation. After proper validation of specific criteria, pre-RLT PSMA PET/CT scan could serve as a baseline for subsequent assessments.

The previously cited PPP criteria have been applied to PSMA-RLT response assessment by some authors, obtaining substantial interobserver agreement and resulting in being prognostic for OS.^68^ PPPs are based on enumerating lesions, which is very advantageous in oligometastatic or limited systemic disease settings.

In cases of high tumor burden at baseline, a substantial risk is the misinterpretation of new small lesions in the post-treatment PSMA PET/CT. For this purpose, in a consensus meeting among PCa experts, the panelists agreed on requiring an increase of total tumor volume >30% to classify a polymetastatic PCa as ‘non-responder’, while the appearance of >2 new focal areas is a sufficient element for low-burden diseases.^51^

Providing also a clear and standardized definition of PR, Gafita *et al.*^69^ developed novel RECIP 1.0 criteria (Table 3), based on PSMA-positive total tumor volume (PSMA-VOL) and appearance of new lesions.

**Table 3.** RECIP 1.0 criteria.

Lesions	PSMA-VOL_PR (decrease >30%)	PSMA-VOL decrease <30%	PSMA-VOL increase <20%	PSMA-VOL_PD (increase >20%)
No new lesions	RECIP-PR	RECIP-SD	RECIP-SD	RECIP-SD
New lesions	RECIP-SD	RECIP-SD	RECIP-SD	RECIP-PD

PD, progressive disease; PR, partial response; PSMA-VOL, prostate-specific membrane antigen-positive total tumor volume; SD, stable disease.

PSMA-VOL was obtained through a semiautomatic segmentation software, using SUV of 3 as a threshold for bone lesions.

RECIP 1.0 criteria categorize complete response, PR, PD, and SD.

PD according to RECIP 1.0 (RECIP-PD) requires both the occurrence of new lesions and a defined minimum increase in tumor volume; for example, the appearance of new lesions and a concomitant decrease in total disease burden is classified as SD (RECIP-SD). Conversely, PR (RECIP-PR) requires both the absence of new lesions and a substantial decrease (>30%) of PSMA-positive tumor volume. These requisites provide an adequate assessment of heterogeneous response.

RECIP 1.0 also combines PSMA imaging data with PSA levels, defining a composite response classification (Table 4).

**Table 4.** PSA + RECIP composite response assessment according to RECIP.

Criterion	Definition
Response	(a) PSA ⩾ 50% decrease or (b) RECIP-PR/complete response
Progression	(a) PSA ⩾ 25% increase or (b) RECIP-PD

PD, progressive disease; PR, partial response; PSA, prostate-specific antigen.

The second version of PROMISE encourages reporting tumor volume changes according to PPP or RECIP, integrating molecular imaging parameters, that is, tumor volume and the occurrence of new lesions.^70^

Various studies showed that PSMA PET/CT biomarkers, mostly PSMA-VOL, can outperform PSA in response assessment and can predict OS.^70–72^

Grubmüller *et al.* retrospectively assessed the response to ^177^Lu-PSMA therapy by performing PSMA PET/CT scans before and after RLT and calculating total tumor volume to apply mPERCIST criteria. They found concordance between PSA and total tumor volume response, both associated with OS, contrary to the morphological evaluation based on RECIST 1.1.^71^

Rosar *et al.* confirmed the association between PSMA-VOL-based response (assessed using PERCIST 1.0 criteria) and OS in 66 patients treated with ^177^Lu-PSMA and undergoing a PSMA PET/CT scan before the first and after the second cycle of RLT. On univariate analysis, both biochemical and molecular response assessments were significantly associated with OS. Furthermore, they reported total lesion PSMA as an independent predictor of survival on a multivariable analysis, showing its superiority to PSA-based response assessment.^72^

Seifert *et al*., considering total tumor volume a negative prognostic factor and SUVmean a positive one, proposed a new prognostic biomarker, total lesion quotient (PSMA-TLQ = PSMA-VOL/SUVmean), to avoid possible neutralization of the two biomarkers. The authors evaluated the potential prognostic value of baseline PSMA PET/CT parameters in 110 mCRPC patients undergoing ^177^Lu-PSMA therapy. PSMA-VOL and PSMA-TLQ were found to be significant negative predictors of survival, while PSA was not.^70^

Other authors evaluated the efficiency of the diverse PSMA-based criteria and compared them with the current response assessment modalities.^73,74^

Gafita *et al.* compared the prognostic value and the inter-reader reliability of RECIST 1.1, aPCWG3, aPERCIST, PPP, and RECIP 1.0. In their work, patients classified as having PD and non-PD patients according to RECIST 1.1 criteria had a similar risk of death, while patients with PD according to RECIP 1.0 had the highest risk of death. PD according to RECIP 1.0 and PPP had a comparable association with OS, which was significantly superior to that of RECIST 1.1. RECIP 1.0 and PPP also had the best agreement among readers compared to the others.^75^

A potential false-negative finding on PSMA PET/CT is the occurrence of neuroendocrine differentiation, a negative prognostic event that can affect advanced disease and has been demonstrated to cause PSMA suppression.^76^

The use of additional assessment with a [^18^F]FDG PET/CT could be advantageous in cases of suspected de-differentiation,^77,78^ but proper indications or timing to perform a [^18^F]FDG PET/CT scan are not established yet.

Emmett *et al* performed both PSMA PET/CT and [^18^F]FDG PET/CT for screening before ^177^Lu-PSMA RLT and for response evaluation in 14 men, defining 3 patterns of progression or response: pattern 1 with no new sites and reduction or absent PSMA uptake at all previous sites; pattern 2 with low or negative PSMA expression progression, and/or FDG-positive-PSMA-negative new lesions; pattern 3 with new sites of high PSMA expression. The authors concluded that evaluating PSMA expression phenotype is fundamental for the definition of subsequent strategies for mCRPC patients.^79^

Phase III trial PSMAfore (ClinicalTrials.gov Identifier: NCT04689828) was recently presented at the 2023 ESMO annual meeting, displaying the superiority of ^177^Lu-PSMA-617 over ARPI in terms of radiographic PFS (rPFS), in taxane-naïve patients with mCRPC and PSMA-positive lesions.^80^ The ENZA-p trial, discussed at the same meeting, compared Enzalutamide plus ^177^Lu-PSMA with enzalutamide alone, resulting in a loger PSA-PFS and rPFS for patients treated with the combination therapy. In the ENZA-p trial, for the first time in a clinical trial, a PSMA PET/CT was performed *ad interim*, and RLT was subsequently continued only in cases of PSMA-avid disease.^81^

## Conclusion

The heterogeneity of mCRPC makes response assessment challenging, potentially requiring distinguishing between PSA-secreting and non-secreting disease, PSMA-expressing and non-expressing lesions, flare phenomena, and de-differentiation.

PSMA PET/CT is accurate and capable to provide early, precise, and informative data that could be used for assessing treatment response in mCRPC patients, in combination with other molecular, biochemical, and clinical parameters to overcome the limitations of each method.

Proper validation of existing or novel frameworks is needed and the real-world impact of molecular imaging data on clinical management of advanced PCa is still to be explored.

## Declarations

**Ethics approval and consent to participate:** Not applicable.

**Consent for publication:** Not applicable. The images included are original and are part of an observational study (244/2016/O/Oss): the two patients signed a study specific informed consent.

**Availability of data and materials:** Not applicable.
